# Temperature Dependence of the Resonant Magnetoelectric Effect in Layered Heterostructures

**DOI:** 10.3390/ma10101183

**Published:** 2017-10-16

**Authors:** Dmitrii A. Burdin, Nikolai A. Ekonomov, Dmitrii V. Chashin, Leonid Y. Fetisov, Yuri K. Fetisov, Mikhail Shamonin

**Affiliations:** 1 Research and Education Center “Magnetoelectric Materials and Devices”, Moscow Technological University (MIREA), 119454 Moscow, Russia; phantastic@mail.ru (D.A.B.); economov@list.ru (N.A.E.); chashin@mirea.ru (D.V.C.); fetisovl@yandex.ru (L.Y.F.); fetisov@mirea.ru (Y.K.F.); 2 East Bavarian Centre for Intelligent Materials (EBACIM), Ostbayerische Technische Hochschule (OTH) Regensburg, D-93053 Regensburg, Germany

**Keywords:** composite structure, magnetoelectric effect, linear, nonlinear, magnetostriction, piezoelectric, acoustic quality factor, temperature dependence, magnetoelectric heterostructure

## Abstract

The dependence of the resonant direct magnetoelectric effect on temperature is studied experimentally in planar composite structures. Samples of rectangular shapes with dimensions of 5 mm × 20 mm employed ferromagnetic layers of either an amorphous (metallic glass) alloy or nickel with a thickness of 20–200 μm and piezoelectric layers of single crystalline langatate material or lead zirconate titanate piezoelectric ceramics with a thickness of 500 μm. The temperature of the samples was varied in a range between 120 and 390 K by blowing a gaseous nitrogen stream around them. It is shown that the effective characteristics of the magnetoelectric effect—such as the mechanical resonance frequency *f*_r_, the quality factor *Q* and the magnitude of the magnetoelectric coefficient *α*_E_ at the resonance frequency—are contingent on temperature. The interrelations between the temperature changes of the characteristics of the magnetoelectric effect and the temperature variations of the following material parameters—Young’s modulus *Y*, the acoustic quality factor of individual layers, the dielectric constant *ε*, the piezoelectric modulus *d* of the piezoelectric layer as well as the piezomagnetic coefficients *λ*^(n)^ of the ferromagnetic layer—are established. The effect of temperature on the characteristics of the nonlinear magnetoelectric effect is observed for the first time. The results can be useful for designing magnetoelectric heterostructures with specified temperature characteristics, in particular, for the development of thermally stabilized magnetoelectric devices.

## 1. Introduction

Magnetoelectric (ME) effects in composite structures comprised of ferromagnetic (FM) and piezoelectric (PE) layers manifest as a change of the polarization of the structure *P* under the influence of an external magnetic field *H* (direct effect) or a change of the structure’s magnetization *M* with an external electric field *E* (converse effect) [1]. ME effects arise from the mechanical coupling across the interface of the constitutive layers as a result of a fusion of the magnetostriction (MS) of the FM layer, the PE effect in the PE layer, and the acoustic properties of the structure as a whole [2]. At room temperature, the magnitude of the ME effect in composite layered structures consisting of materials with high magnetostriction coefficient *λ* (NiZn- and Co-based ferrites; metals Ni and Co; alloys like galfenol and terfenol; amorphous alloys, etc.) and materials with a large piezoelectric modulus *d* (lead zirconate titanate (PZT)-based ceramics, lead magnesium niobate-lead titanate (PMN-PT), AlN, LiNbO3, GaAs, PE polymers, etc.) exceeds the ME effect in natural crystals Cr_2_O_3_, FeBO_3_, etc. [3,4] by several orders of magnitude. This fact makes ME effects in composite structures very promising for developing novel microelectronic and information processing devices such as high-sensitivity magnetic field sensors [5,6,7], tunable radio signal processing devices [8,9,10], novel magnetic memory elements [11,12,13], etc.

One of the less investigated issues—both from the point of view of physics and real-world applications—is the analysis of the temperature characteristics of ME effects. It is obvious that the temperature dependence of magnetic, dielectric and mechanical properties of individual layers of the structure as well as the efficiency of the layer bonding should lead to temperature dependences of the characteristics of the ME effect. Similar considerations are also important for PE vibration energy harvesters, where the output power as a function of temperature can be predicted by the change to the PE coupling coefficient, which is proportional to the PE constant and inversely proportional to the square root of the dielectric constant [14]. Mostovoy et al. explained the temperature dependence of the magnetoelectric coupling in Cr_2_O_3_ by nonrelativistic exchange interactions and spin fluctuations using a combination of ab initio methods, symmetry arguments, and Monte Carlo simulations [15].

In recent years, preliminary experimental studies of the temperature characteristics of ME effects in structures of various compositions and geometries have been reported in the literature.

Zhang et al. investigated the direct ME effect in two-layer structures with FM layers from polycrystalline or single crystalline lanthanum-strontium manganite (LSMO) and PE layers of lead zirconate titanate (PZT) ceramics bonded together by an adhesive [16]. It was shown that in the temperature range between 80 and 300 K the efficiency of the ME interaction *α*_E_ in polycrystalline samples was approximately equal to 80 mV/(Oe × cm) and it was weakly dependent on the temperature. For single crystalline samples, *α*_E_ increased from 80 mV/(Oe × cm) to 120 mV/(Oe × cm) with increasing temperature. The authors attributed this effect to the change in the magnetostriction of LSMO. Li et al. investigated the LSMO-BTiO_3_ film samples, fabricated by laser sputtering, and observed a significant decrease in the ME effect with increasing temperature in the range between 250 and 350 K [17]. This effect also depended on the thickness ratio of the constitutive layers. Vaz et al. investigated LSMO-PZT structures, fabricated by molecular epitaxy and magnetron sputtering methods, and observed a strong temperature dependence of the converse ME effect in the range between 100 and 250 K with a maximum at the phase transition temperature of about 180 K [18].

Fang et al. studied the temperature dependence of the efficiency of ME interactions in three-layer structures with a PE PZT layer and FM layers of terfenol, nickel, or an amorphous FeBSiC (Metglas) alloy fabricated by epoxy adhesive bonding [19]. It was shown that in the temperature range between 25 and 80 °C the resonance frequency of Metglas-PZT-Metglas and Ni-PZT-Ni structures decreased by approximately 4% with growing temperature, the resonance frequency of the Terfenol-PZT-Terfenol structures did not change, and the ME effect in the Metglas-PZT-Metglas and Ni-PZT-Ni structures monotonically several-fold declined with increasing temperature. The authors explained the change in the resonance frequency as a result of temperature change in the rigidity of the layers, and the decrease in the ME effect by a change in the piezomagnetic modulus of the FM layers.

Gutierrez et al. investigated three-film structures with a magnetic layer of the amorphous magnet Vitrovac 440 (FeNiMoSiB) and PE layers of a PE polymer (polyvinilidene flouride, PVDF), fabricated by adhesive bonding [20]. At the acoustic resonance frequency of the structures, a sharp drop in the efficiency of the ME interaction from 80 V/(Oe × cm) to approximately 2 V/(Oe × cm) was observed with an increase in temperature from 20 to 80 °C, which was attributed to the degradation of PE properties of PVDF.

Burdin et al. investigated the temperature characteristics of the resonant ME effect in structures with ferromagnetic Ni layers and PE layers of PMN-PT single crystals or PZT ceramics [21,22,23,24]. It was shown that in PMN-PT-Ni structures fabricated by adhesive bonding, the resonance frequency of the flexural and planar resonance modes of the structure reduces by 6–10% with the temperature growth from 220 to 340 K [21]. The ME interaction efficiency depended weakly on the absolute temperature *T* up to 270 K, and then decreased sharply. This fact was attributed to the softening of the adhesive bonding layers.

In the disk Ni-PZT resonators, the type of the temperature characteristics depended on the fabrication method [22,23,24]. In a monolithic structure made by the electrolytic deposition of Ni on a PZT disk, a non-monotonic change in the resonance frequency with a local minimum at *T* = 270 K and a monotonic decrease in efficiency with increasing *T* were observed. These effects were explained by the difference in the temperature expansion coefficient of these materials, leading to their static deformation. In the case of adhesively bonded PZT and Ni layers, a significant reduction of the resonance frequency and the non-monotonic behavior of the ME interaction efficiency with increasing temperature, caused by softening of adhesive bond strength were observed.

The temperature characteristics of the ME effect in structures of different compositions—in particular langatate (LGT)-Ni, LGT-Metglas, PZT-Ni and PZT-Metglas—in the temperature range between 200 and 400 K were examined in reference [24]. The constitutive layers had a thickness of between 20 μm and 0.5 mm. It was shown that the resonance frequency depends weakly on temperature in structures containing layers of thermostable Metglas, and the magnitude of the effect usually decreases with increasing temperature. The possibility of the temperature stabilization of structures by the appropriate selection of the properties of its constituent layers was demonstrated.

Ye et al. observed the non-monotonic variation (existence of a local maximum) of the ME coefficient with increasing temperature in a composite structure comprising a PZT layer sandwiched between two Metglas layers [25].

Zhang and Gao developed a nonlinear dynamic hysteretic theoretical model of magnetoelectric effect in tri-layered (Terfenol-D-PMN-PT-Terfenol-D and Terfenol-D-PZT-Terfenol-D) composites based on a nonlinear constitutive relation for magnetostrictive material and a linear PE model for PE material [26]. They have found that the initial temperature had little effect on the induced electric field in the low-magnetic-field region, but a significant effect on the induced electric field in the high-magnetic-field region.

It was shown that it is possible to thermally stabilize the ME effect and eliminate the pyroelectric effect in composite multilayer BaTiO_3_-Ni structures by the choice of the crystallographic orientation of neighboring PE layers [27].

The temperature dependence of the ME effect in a PZT-Metglas structure was investigated in reference [28]. The ME coefficient monotonically increased with the temperature in the range from −50 to 50 °C.

Hitherto, the ME effect has been experimentally studied in structures of different compositions and it has been shown that the temperature variation significantly affects the ME coefficient and the acoustic resonance frequency of a composite structure. Unfortunately, comprehensive analysis of the influence of different structural parameters on the temperature dependences of the ME effect has not been performed yet. As a rule, the explanation of the observed changes in the ME coefficient is associated with the change of the PE coefficient of the PE layer of the structure and the decrease of the magnetostriction of the FM layer as well as with the variation of the mechanical bonding strength between the layers (softening of the adhesive layer with the increasing temperature). While the temperature dependences of the PE modulus of materials are known from the literature, there is practically no data available for the piezomagnetic (PM) modulus of the FM layer. The authors are aware of a single paper [19], in which the necessary measurements of the field dependences for Metglas, nickel and Terfenol-D were carried out in the range between 25 and 80 °C, which is insufficient for explaining the non-monotonic behavior of the temperature dependence of the ME coefficient of a PZT-Metglas structure from reference [25] as well as the monotonic growth of *α*_E_ in the temperature range between −50 and +40 °C, described in reference [28]. The analysis of the contribution of the PE layer is also insufficient, as it does not take into account the temperature change of the dielectric constant and does not compare the characteristics of structures with different constitutive materials of the PE layer.

Therefore, it is necessary to conduct a comprehensive study on structures with different materials composing the PE and FM layers in order to elucidate the factors that determine the origin of the temperature dependences of their magnetoelectric characteristics. To do this, it is required to determine the behavior of various parameters of the constitutive layers (piezomagnetic coefficient of the FM layer, permittivity and piezoelectric modulus of the PE layer, Young’s moduli) over a wide temperature range. A comparison between structures with different compositions may allow the assessment of its influence on the ME effect.

The following Section 2 of the present paper describes the samples of composite structures and the experimental methods for studying the temperature characteristics of the ME effect in composite structures. In the third section, the temperature characteristics of the magnetostrictive and PE layers of the structures influencing the ME effect are given. It contains the results of measurements of the temperature characteristics of the linear ME effect in structures with a small excitation field. Section 3 is devoted to the theoretical analysis and explanation of measurement results. Section 4 describes the study of the temperature characteristics of the nonlinear ME effect in composite structures and provides an interpretation of the results. The conclusion section summarizes the main results of this paper and provides recommendations for their real-world application.

## 2. Experimental

### 2.1. Samples

Investigations of thermal characteristics of the ME effect were performed on two-layer FM-PE structures with layers of different materials and different thicknesses. For fabrication of PE layers, two widely used materials with very different properties were employed: a PE ceramics with the composition (Pb_0.95_Sr_0.05_)(Zr_0.53_Ti_0.47_)O (type PZT 5, manufactured by ELPA R&P Company, Zelenograd, Russia) [29] and an X-cut monocrystalline langatate material with the composition La_3_Ga_5.5_Ta_0.5_O_14_ (LGT) (manufactured by FOMOS-materials, Moscow, Russia) [30]. The main electrical and acoustic parameters of these materials at room temperature are given in Table 1.

The PE ceramic PZT-5 has a sufficiently high piezoelectric module *d*_31_ ≈ 200 pC/N and a large dielectric constant of *ε* ≈ 1200, but a low acoustic quality factor *Q* ≈ 100 and a small Young’s modulus *Y* ≈ 6.2 × 10^10^ Pa. Langatate crystals, unlike ceramics, have a small PE modulus *d*_11_ = 5.1 pC/N, a low dielectric constant *ε*_11_ = 20, but they possess a high acoustic quality factor *Q* ≈ 10^4^ and a high Young’s modulus *Y* ≈ 26 × 10^10^ Pa [31]. The ratio *d*/ε for PE ceramics and single crystals is ~0.1 and ~0.25, respectively, which enables a large ME effect. The thickness of the PZT layers was 0.45 mm, the thickness of the langatate layers was 0.5 mm. Ag electrodes with the thickness of approximately 3 μm were deposited on the surface of the PZT layers by the burning-in method. The PZT layers were polarized by heating to 100 °C and applying a constant voltage of 500 V for 2 h. The Ag electrodes with thickness of approximately 1 μm were sputtered on the surface of the LGT samples, the polarization of the langatate crystals was not necessary.

The magnetostrictive layers were made of common materials with high magnetostriction: the ribbon of the amorphous ferromagnetic alloy Metglas 2605S3A with the composition FeBSiC [32] and a thickness of 20 μm or a polycrystalline nickel ribbon with a thickness of 50 μm to 200 μm. The main magnetic and mechanical parameters of the FM layers are also given in Table 1. Both materials have a sufficiently high saturation magnetostriction |*λ*_S_| ≈ (20–30) × 10^−6^. Note that the magnetostriction of Ni is negative. The amorphous alloy Metglas is saturated in small magnetic fields *H*_S_ ≈ 60 Oe, which provides a high value of the piezomagnetic and ME coefficients. The mechanical quality factor of both FM materials is low *Q* ~ 10^2^, and the Young’s modulus has an intermediate value of *Y* ≈ (10–20) × 10^10^ Pa.

Rectangular plates (slabs) with a length of 20 mm and a width of 5 mm were cut out of the FM and PE materials. Next, FM and PE layers of different thicknesses were bonded together under pressure with a quick-drying adhesive (LOCTITE^®^ 406™, Henkel AG & Co. KGaA, Munich, Germany) that retained its mechanical properties in the temperature range from −100 °C to +100 °C. The thickness of the bonding layer did not exceed approximately 5 μm. The composition and parameters of the fabricated bilayer structures are shown in Table 2. The index“_T” denotes samples with a thicker layer of nickel.

### 2.2. Methods of Measurement

The quality factor and Young’s modulus of PE layers were measured using the series resonance method [33]. For this purpose, a resistance *R* of 1 kΩ was connected in series with the sample, a voltage with the fixed amplitude of 1 V at frequency *f* was applied to the sample and the voltage at the resistance was recorded as the frequency was varied. The quality factor of the PE-layer was calculated from the width of the resonance curve at the −3 dB-level, and the value of the Young’s modulus was calculated from the longitudinal resonance frequency using the known formulas [34].

The quality factor and Young’s modulus of FM layers were measured by the method of mutual kinetic impedance [35]. For this purpose, two electromagnetic coils with 100 turns each were wound on the FM sample. An alternating current with the frequency *f* and the amplitude of 1 mA was driven through the excitation coil and the voltage induced in the receiver coil was recorded as the frequency was varied. As in the previous case, the acoustic quality factor of the FM layer was calculated from the width of the resonance curve at the level of −3 dB and the value of Young’s modulus was determined from the longitudinal resonance frequency.

Measurements of the acoustic parameters of the layers were performed in the temperature range between 170 and 370 K using the experimental setup described below.

The magnetostriction of the FM layers *λ*(*H*) was measured in the magnetic field range up to 2 kOe by the strain-gauge method with the help of a foil strain gauge fixed onto the surface of the sample. To increase the accuracy, the temperature of the FM sample during the measurements was stabilized by a Peltier element with an accuracy *δT* of approximately ±0.1 K. The uncertainty of measuring the magnetostriction was *δλ* ≈ ±0.5 × 10^−6^.

Magnetization curves *M*(*H*) of FM layers were measured in the magnetic-field range up to 2 kOe and in the temperature range between 140 and 380 K using a vibrating sample magnetometer LakeShore 7400 (Lake Shore Cryotronics, Inc., Westerville, OH, USA; located at Department of Magnetism, M.V. Lomonosov Moscow State University).

The electrical capacitance and dielectric losses tan*δ* of PE layers were measured using an RLC-meter AM-3026 (Eliks, Moscow, Russia) in the frequency range from 20 Hz to 5 MHz. The value of the relative permittivity of PE layers *ε* was calculated from the formula for a flat capacitor. Measurements of the dielectric parameters of the layers were performed in the temperature range between 170 and 370 K using a thermostat.

**Figure 1:**
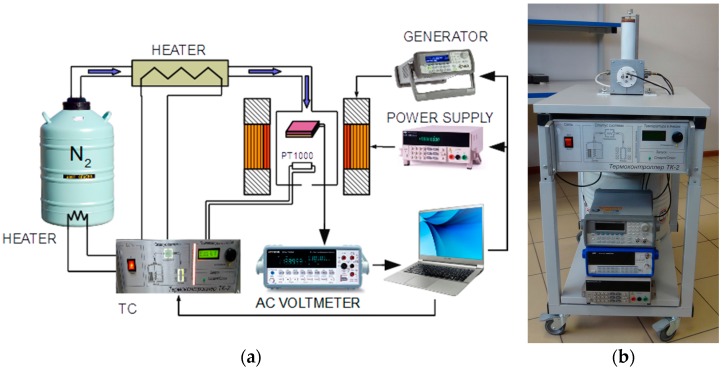
Block diagram of the apparatus for the investigation of temperature characteristics of the magnetoelectric (ME) effect in composite structures (**a**) and its appearance (**b**). TC denotes the temperature controller.

To investigate the temperature characteristics of the ME effect, an automated apparatus was developed, the block diagram of which is shown in Figure 1a and its appearance is given in Figure 1b. A composite structure was placed between the poles of an electromagnet, which imposed a uniform constant magnetic field *H* up to 2 kOe. The magnetic field was directed along the long side of the structure. Simultaneously, an alternating magnetic field *h*_0_cos(2π*ft*) with the amplitude *h*_0_ of up to 10 Oe in the frequency range between 20 Hz and 200 kHz was applied in the same direction using modulating coils fed by a signal generator (Agilent 33210a, Agilent Technologies, Inc., Santa Clara, CA, USA). The voltage *u*(*t*) generated by the structure was measured with a digital voltmeter (AKIP-2401, AO “PriST”, Moscow, Russia) with the input resistance of 10 MΩ. This setup made it possible to obtain the dependence of the amplitude *u*_0_ of the generated voltage on *H*, *h*_0_ and *f*. The frequency spectrum of the voltage was obtained using the fast Fourier transform algorithm.

To measure the temperature characteristics, the structure was placed in a thermally insulated cell made of Teflon. A stream of nitrogen gas was pumped through the measurement cell. This gas stream cooled or heated the sample to the desired temperature. The flow rate of nitrogen was adjusted by means of an evaporator placed inside the Dewar vessel while the flow temperature was controlled by a second heater. Such a system made it possible to avoid the build-up of a condensate on the surface of the sample at low temperatures. The temperature of the evaporator, heater and sample was monitored using platinum resistance thermometers Pt-1000 (HEL-711-U-0-12-00, Honeywell International Inc., Morristown, NJ, USA). The system was controlled by a temperature controller and a specifically designed program in the LabVIEW environment (National Instruments, Austin, TX, USA). The apparatus enables one to carry out the measurements in the temperature range between 150 and 390 K with the temperature keeping accuracy *δT* of approximately ±0.2 K for an arbitrary preset time dependence of the temperature.

A specifically developed LabVIEW application was used to determine the resonant frequency *f*_r_, and the quality factor of the structure, *Q* = *f*_r_/Δ*f* (where Δ*f* is the curve width at the −3 dB-level), for each temperature *T*. The obtained data allowed one to construct the *f*_r_(*T*)- and *Q*(*T*)-dependences. The same application determined the value of the voltage *u*_0r_ at the resonance frequency *f*_r_ and plotted the dependence of the ME conversion coefficient *α*_E_(*T*) on the temperature.

## 3. Results and Discussion

### 3.1. Temperature Characteristics of Ferromagnetic and Piezoelectric Layers

In order to clarify the mechanisms of the temperature effect on the resonance frequency and the magnitude of the ME effect in composite structures, the dependences of the most important parameters of the FM and PE layers on the temperature were studied separately as a first step. The measured absolute values of the Young’s modulus *Y* and the quality factor *Q* of the employed FM and PE layers at the room temperature of 290 K are given in Table 1.

**Figure 2:**
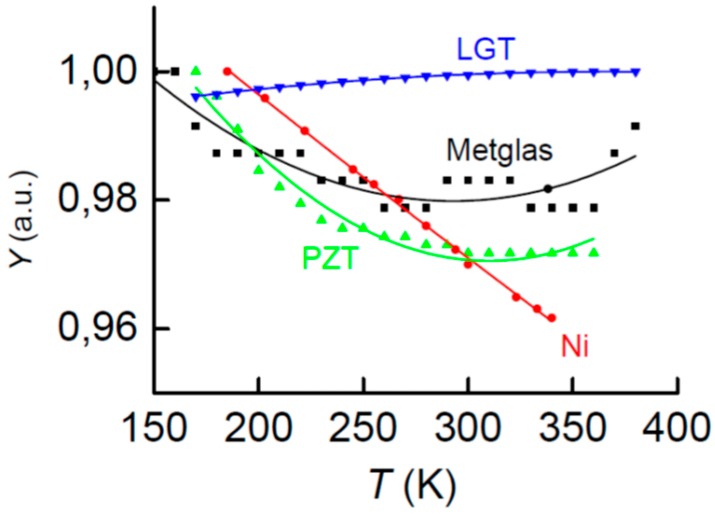
Temperature dependences of the normalized Young’s modulus *Y* of PE and FM layers. Solid lines represent the approximation of data by a second-order polynomial.

Figure 2 shows the Young’s modulus *Y* of the constitutive layers as a function of temperature from 160 to 370 K. For convenience of comparison, the values were normalized to the maximum value of a particular material. It can be seen that the Young’s modulus of the langatate grows by not more than 0.2% with increasing temperature and the rigidity of all other materials decreases by not more than 4% with increasing temperature.

**Figure 3:**
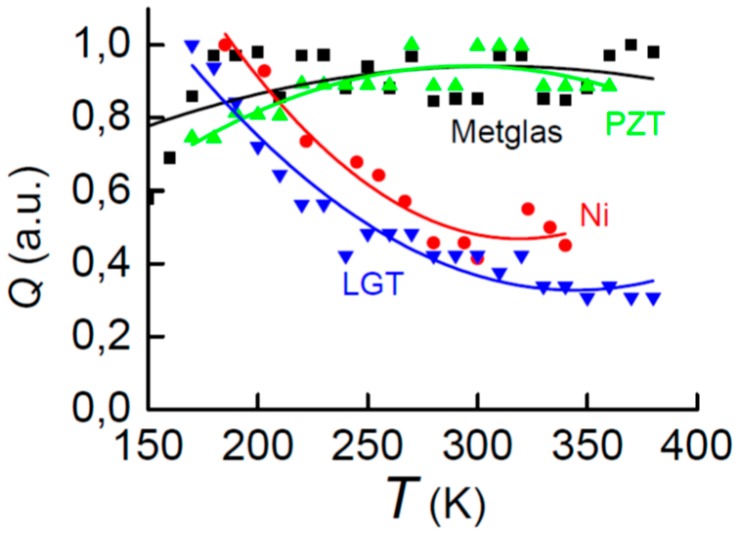
Temperature dependences of the normalized quality factor *Q* of PE and FM layers. Solid lines represent the approximation of data by a second-order polynomial.

Figure 3 presents the normalized quality factor for all layers as a function of temperature. It can be seen that the quality factor of the PZT and Metglas layers vary by less than approximately 20%, while the quality factor of the Ni and langatate layers monotonically decreases by two- or threefold, respectively, with increasing temperature.

**Figure 4:**
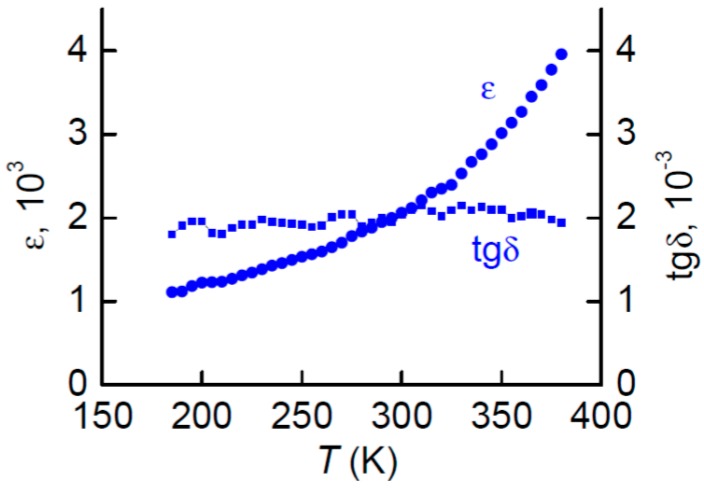
Temperature dependences of the relative dielectric permittivity and dielectric losses for the lead zirconate titanate (PZT) material.

Figure 4 depicts the measured dependences of the relative permittivity *ε* and the dielectric losses (tan*δ*) of the PZT layer on the temperature. It is seen that *ε* monotonically grows from 1000 to 4000 with *T* increasing from 160 to 380 K, while tan*δ* practically does not change. The langatate crystal belongs to the class of thermostable dielectrics [36], its dielectric permeability and losses in the investigated temperature range remain practically constant and were equal to their values at room temperature (see Table 1).

**Figure 5:**
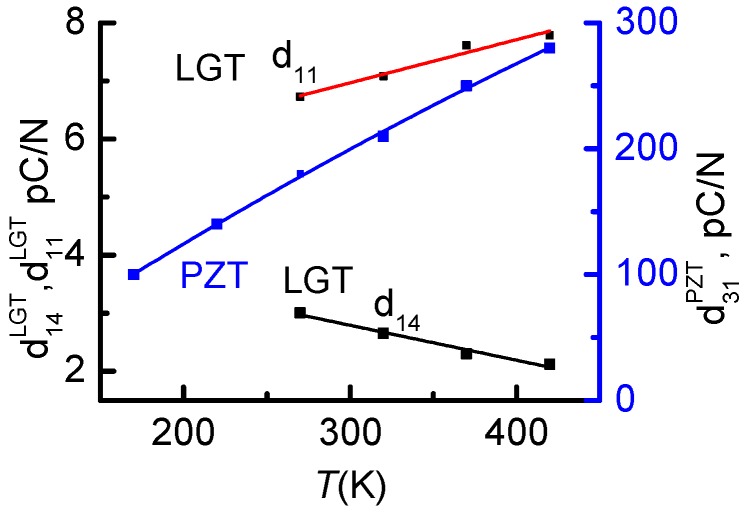
Temperature dependences of piezoelectric moduli for La_3_Ga_5.5_Ta_0.5_O_14_ (LGT) and PZT materials [36,37,38].

Figure 5 shows the temperature dependences of PE modules for PZT and LGT materials using the data published in [36,37,38]. The PE modulus *d*_14_ of the LGT material monotonically decreases by 25% from 3 to 2.2 pC/N with the temperature growth from 270 to 420 K, while the *d*_31_-modulus of the PZT material, on the contrary, monotonically increases threefold (from 100 to 300 pC/N) when the temperature increases from 200 to 420 K. As explained in reference [39], the growth of PE coefficients *d* with increasing temperature can be attributed to the following two mechanisms: (1) the extrinsic PE response is enhanced because lower activation energy is required for transition of domains from one minima state to another; (2) according to thermodynamic theory calculations the PE response of PZT in the single domain state increases with growing temperature as well.

**Figure 6:**
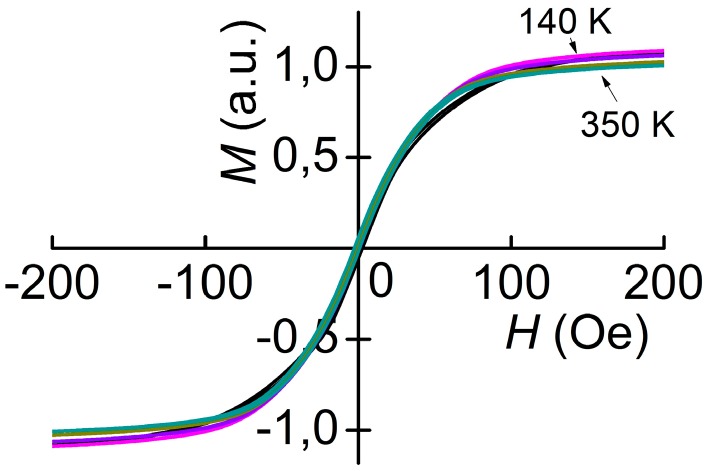
Magnetization curves of the Metglas layer for different temperatures *T*.

**Figure 7:**
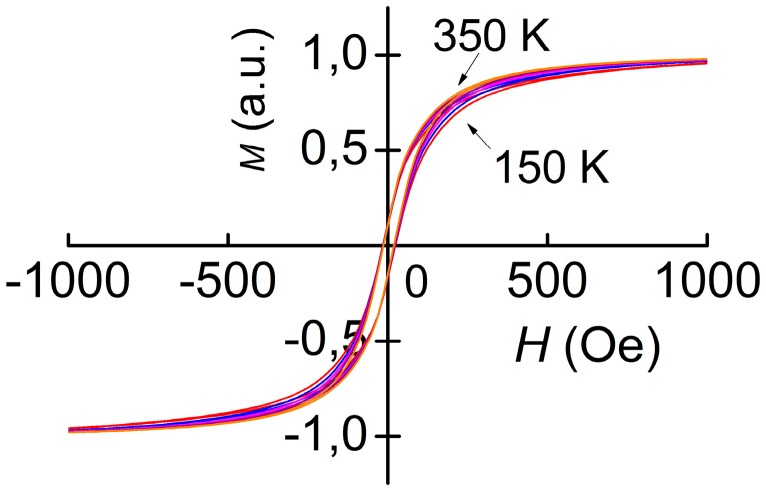
Magnetization curves of the Ni layer for different temperatures *T*.

Figure 6 and Figure 7 display the magnetization curves of FM Metglas and Ni layers, respectively, measured at different temperatures. The value normalized to the magnetization at room temperature is plotted on the vertical axis on the graphs. From Figure 6 it is seen that Metglas is saturated already at *H*_S_ ≈ 60 Oe, the width of the loop is less than 1 Oe, an increase in temperature from 140 to 350 K leads to a decrease in the saturation magnetization of the sample by 7%. The nickel layer is saturated in fields *H*_S_ ≈ 500 Oe. The saturation magnetization of nickel is practically constant in the indicated temperature range. The loop width is about 10 Oe. For nickel, the shape of the magnetization loop slightly changes with increasing *T*.

**Figure 8:**
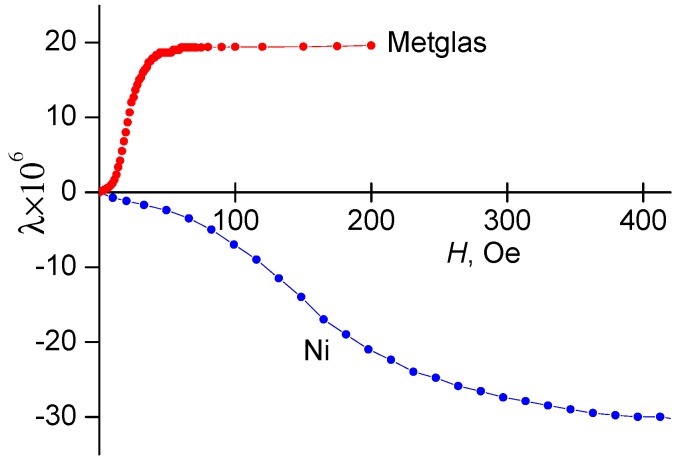
Dependences of magnetostriction *λ* for Metglas and Ni slabs on magnetic field *H* at room temperature.

**Figure 9:**
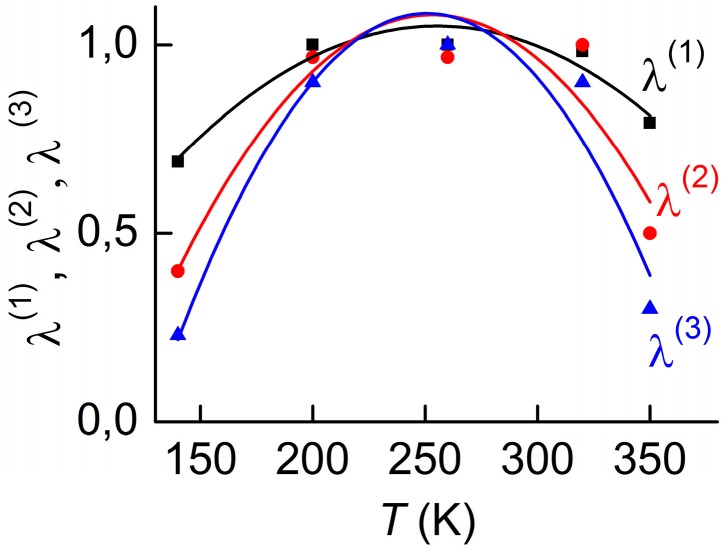
Temperature dependences of derivatives of magnetostriction coefficient on magnetic field for a Metglas layer. Solid lines represent the approximation of data by a second-order polynomial.

**Figure 10:**
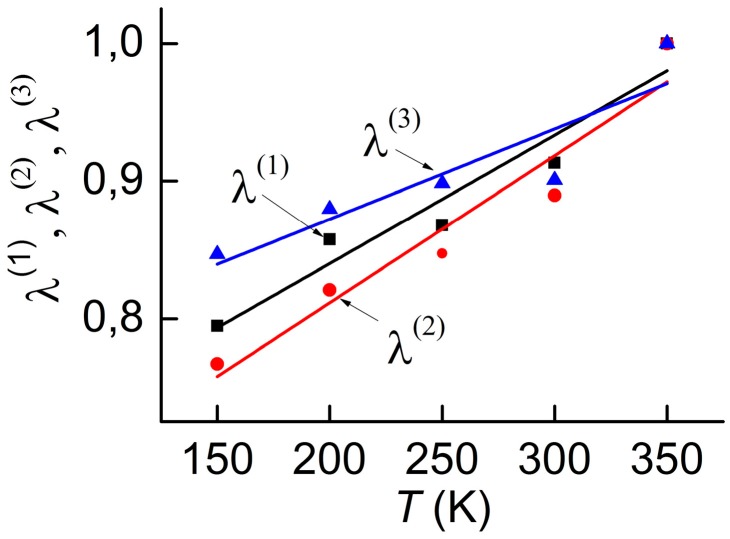
Temperature dependences of derivatives of magnetostriction coefficient on the magnetic field for a Ni layer. Solid lines represent the approximation of data by a straight line.

Figure 8 displays the measured dependences of the magnetostrictive deformation of Metglas and Ni layers on the magnetic field *H* at room temperature. It is known that the magnitude of the linear ME effect is proportional to the first derivative $\lambda^{\left(1\right)}=\partial \lambda /\partial H$, and the magnitude of the nonlinear effects are proportional to the second $\lambda^{\left(2\right)}=\partial^{2}\lambda /\partial H^{2}$ and third derivatives $\lambda^{\left(3\right)}=\partial^{3}\lambda /\partial H^{3}$ of the magnetostriction with respect to the field [40]. Therefore, for the purposes of the present work, it is necessary to know the dependence of these derivatives on the temperature. The experimental setup did not allow measurement of the *λ*(*H*)-curves in the entire temperature range of interest. Therefore, temperature dependences of the derivatives were obtained by an indirect method, using the relationship between the magnetostriction *λ* of the FM layer with the magnetization *M* [41]. Using the *λ*(*H*)-curves measured at different *T*, the dependences $\lambda (H)\approx \kappa M^{2}(H)$ were calculated for different *T*, and then the field dependences of the derivatives $\lambda^{\left(1\right)}(H)$, $\lambda^{\left(2\right)}(H)$, and $\lambda^{\left(3\right)}(H)$ were found by numerical differentiation. On each of these curves, a maximum was determined at the corresponding field *H*_m_, and the dependences of the maximum values of the derivatives on *T* were derived. The dependencies $\lambda^{\left(1\right)}(T)$ at *H*_m1_ = 8 Oe, $\lambda^{\left(2\right)}(T)$ at *H*_m2_ = 0 Oe and $\lambda^{\left(3\right)}(T)$ at *H*_m3_ = 4 Oe for the Metglas layer as well as the same dependencies for the Ni layer in the field where the derivatives have their maximum, are shown in Figure 9 and Figure 10, respectively.

It can be seen that the coefficients *λ*^(1)^, *λ*^(2)^ and *λ*^(3)^ for the Metglas layer have approximately the same dependence on the temperature and possess a characteristic maximum in the vicinity of *T* ≈ 250 K. In this case, the overall change of *λ*^(1)^ is about 25%, *λ*^(2)^ changes by 50% and *λ*^(3)^ varies by 75% in the investigated temperature range. It should be noted that for the Metglas material the decrease of *λ*^(1)^ in the temperature range from 300 to 350 K agrees with the data obtained in [19]. The derivatives *λ*^(1)^, *λ*^(2)^ and *λ*^(3)^ of the magnetostriction of nickel grow almost linearly by approximately 20% with an increase of temperature from 150 to 350 K. The temperature dependences of the main parameters of the constitutive layers of composite structures, given in Figure 2, Figure 3, Figure 4, Figure 5, Figure 9 and Figure 10, will be used below to explain the temperature characteristics of the ME effect in these structures.

### 3.2. Temperature Characteristics of the Linear Magnetoelectric Effect

First, let us consider the temperature characteristics of the linear ME effect in the above described structures, measured at *h*_0_ = 0.1 Oe, which is much smaller than the saturation fields of the layers. The main characteristics of the linear ME effect are the frequency of the acoustic resonance of the structure *f*_r_, the quality factor of this resonance *Q* = *f*_r_/Δ*f*, and the value of the ME voltage coefficient α_E_ = *u*/(*hb*_p_) at the resonance frequency. With a linear effect, the amplitude of the ME voltage *u*_0_ is proportional to *h*_0_ and therefore the value of the ME coefficient is independent of the magnitude of the ac field.

Table 3 shows the values of the resonance frequency *f_r_*, the acoustic quality factor *Q*, and the ME coefficient *α*_E_ at the resonance frequency, measured for all structures at room temperature. Resonance frequencies for all samples were in the range of ≈60–100 kHz and were in agreement with the theoretical estimates accordingly to the formulae from reference [34] for the parameter values corresponding to present experiments.

The highest quality factor *Q* ≈ 6000 was observed in samples comprising LGT single crystals and thin FM layers, and the lowest quality factor *Q* ≈ 100 was found in samples with PZT layers and thick Ni layers. The largest ME effect was found in structures with Metglas layers, with the highest value of *α*_E_ = 450 V/(Oe × cm) being observed in the LGT-Metglas structure. The ME coefficient of the LGT-Metglas laminate is significantly larger than the ME coefficient of the PZT-Metglas sample because of the much lower dielectric constant *ε* of the LGT material (see Table 1).

The characteristics of all structures in the temperature range between 170 and 370 K were measured. Since the resonant frequencies *f*_r_ and ME coefficients *α*_E_ for the structures of interest differ significantly by their magnitude, we will compare their normalized values:

(1) $$\delta f=\frac{f_{r}-f_{min}}{f_{min}}\;\mathrm{and}\;\alpha_{E}^{\prime }=\frac{\alpha_{E}}{\alpha_{Emax}},$$

where *f*_min_ is the resonance frequency at the lowest temperature (170 K), *α*_Emax_ is the maximum value of the ME coefficient in the investigated temperature range.

**Figure 11:**
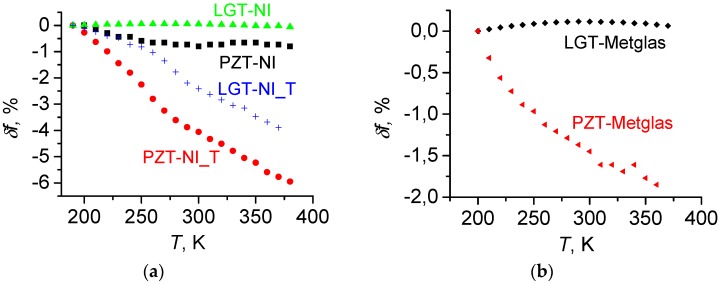
Temperature dependences of the change of the resonance frequency *δf* for ME structures with different compositions comprising Ni (**a**) or Metglas layers (**b**). The index “_T” designates structures with the thickness of a Ni layer of 200 μm.

The obtained dependences of the relative change of the resonant frequency $\delta f_{r}(T)$ for investigated structures are shown in Figure 11a,b. It is seen that the course of the curves is different for different structures. For structures based on a langatate crystal with a thin FM layer, LGT-Ni and LGT-Metglas, the resonance frequency first increases with growing temperature, reaches a maximum at *T* ≈ 300 K, and then declines with increasing temperature. In the investigated temperature range, the change in resonance frequency is insignificant for these samples and is less than 1%. For PZT-based PE ceramic structures, the frequency decreases monotonically and rather strongly with increasing temperature: for the PZT-Ni structure, the frequency decreases by 1%, for the PZT-Metglas the frequency is reduced by 2%. Note that the results obtained agree with temperature dependences of the resonance frequency in reference [19]. An increase in the thickness of the nickel layer from 50 to 200 μm led to a deterioration in the temperature stability of the resonance frequency by an order of magnitude: for the LGT-NI_T structure, the frequency decreased by 4%, and the frequency of the PZT-Ni_T structure declined by 6% with growing *T*.

**Figure 12:**
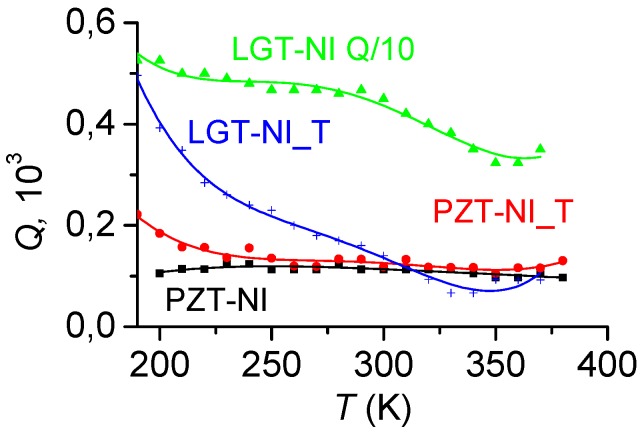
Dependences of quality factor *Q* on temperature. The values for the LGT-Ni structure are scaled down by a factor of 10.

Figure 12 presents the measured temperature dependences of the acoustic quality factor *Q*(*T*) of the structures in the temperature range between 190 and 370 K. It is seen that the quality factor of the structures comprising LGT layers is maximal at low temperatures and it significantly declines with increasing *T*. For the LGT-Ni structure, the *Q*-factor decreases from 5300 to 3000 at 350 K, and for the LGT-Ni_T structure, the quality factor declines from 5000 to 110 at 350 K. The quality factor of structures with layers of PE PZT-ceramics is about 100 and it slightly varies in the investigated temperature range. To the best of our knowledge, dependences of the quality factor of ME structures on the temperature are obtained for the first time. Previously, they have not been taken into account for the analysis of the properties of the ME effect.

**Figure 13:**
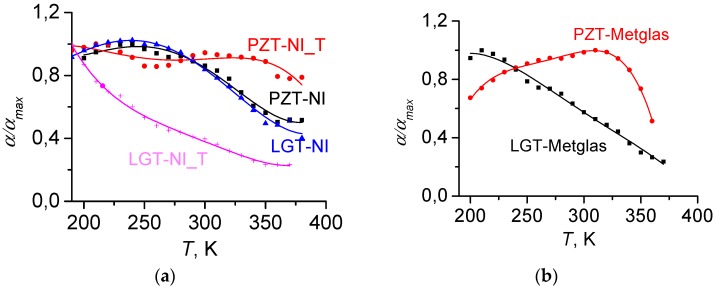
Dependences of the normalized ME coefficient *α*′ on the temperature for structures of different compositions comprising Ni (**a**) or Metglas layers (**b**). The index “_T” designates structures with the thickness of the Ni layer of 200 μm.

Figure 13a,b displays the measured temperature dependences of the normalized ME coefficient *α*′*_E_*(*T*) for the structures under investigation in the temperature range between 190 and 380 K. For all structures, except of PZT-Ni T, the ME coefficient strongly depends on the temperature and, as a rule, decreases with increasing *T*. For structures with LGT layers, the ME interaction efficiency decreases monotonically approximately fivefold with the increase of temperature from 190 to 380 K. Moreover, an increase in the thickness of the Ni layer leads to an even greater decrease of the ME effect. For structures with PZT ceramic layers, the reduction of the ME coefficient with increasing temperature is much smaller. The most stable structure was based on a PZT layer with a thick layer of Ni, where the change of *α*′*_E_* did not exceed 20% in the temperature range between 200 and 380 K. Notice an anomalous increase of the ME coefficient from 0.7 to 1 for the PZT-Metglas structure when the temperature grows from 200 to 320 K. Remarkably, the observed non-monotonic behavior agrees with the results for the Metglas-PZT-Metglas structure obtained in [25].

### 3.3. Discussion of the Linear Magnetoelectric Effect

#### 3.3.1. Temperature Dependence of the Resonance Frequency

The dependence of the longitudinal acoustic resonance frequency of the composite structure of a rectangular shape with the length *L* on its parameters is given by the expression [34]

(2) $$f=\frac{1}{L}\sqrt{\frac{Y}{\rho (1-\gamma )}},$$

where for the two-layer structure the effective values of the Young’s modulus *Y* and the density *ρ* depend on the parameters of the individual layers and their thicknesses:

(3) $$Y=\frac{Y_{m}b_{m}+Y_{p}b_{p}}{b_{m}+b_{p}}\;\mathrm{and}\;\rho =\frac{\rho_{m}b_{m}+\rho_{p}b_{p}}{b_{m}+b_{p}},$$

where *Y*_m_ and *Y*_p_, *ρ*_m_ and *ρ*_p_, *b*_m_ and *b*_p_ are the Young’s modulus, density and thickness of FM and PE layers, respectively, and *γ* ≈ 0.3 is the Poisson’s ratio. Note that the approximation (3) for the effective Young’s modulus is justified, since the constitutive materials have similar Poisson’s ratios and the longitudinal oscillations are considered [42]. Estimations have shown that, because of the temperature expansion, the length *L* of the structure and the density of materials vary within the considered temperature range by less than 0.2%. Therefore, the temperature dependence of the resonance frequency of composite structures arises mainly because of the temperature dependences of the Young’s moduli of the layers. This allows us to explain the dependences in Figure 11.

- In structures with LGT layers and thin FM layers (Metglas or Ni), the resonance frequency weakly depends on the temperature, since the rigidity of the LGT layer insignificantly changes with the temperature (see Figure 3). In this case, an increase of the thickness of the Ni layer, where *Y* decreases with growing temperature, leads to an increase of the relative contribution of Ni to the effective Young’s modulus and to the corresponding decrease in the resonance frequency. This is the case in the experiment (Figure 11a).
- In structures with PZT layers, the reduction of the resonant frequency with growing temperature (see Figure 12) is due to a decrease of the Young’s modulus of the PZT layer (see Figure 2). In this case, the largest reduction of the resonance frequency by 6% occurs in the structure with a thick layer of Ni, whose Young’s modulus also decreases with increasing temperature.
- The growth of the Young’s modulus of the langatate crystal in the temperature range between 200 and 320 K (see Figure 2) allows one to design composite structures with a vanishing temperature coefficient of frequency at room temperature. This can be achieved by the optimal selection of the thickness of the LGT layer and the FM layer, where *Y*_FM_ decreases with increasing temperature. Such an optimization can be used for thermal stabilization of prospective ME devices.

#### 3.3.2. Temperature Dependence of the Quality Factor

To explain the temperature dependences of the quality factor of the ME structures, we express the quality factor of the structure *Q* in terms of the quality factor of the constituitive layers *Q*_e_, *Q*_m_, Young’s moduli of the layers *Y*_e_, *Y*_m_ and the thicknesses of the layers. Denote the length of the structure by *L* and the width of the structure by *w*. Assume that the structure is uniformly and elastically deformed along the longitudinal axis. We represent the total deformation energy of the structure *U* as a sum of the deformation energies of FM (*U*_m_) and PE (*U*_e_) layers:

(4) $$U=U_{m}+U_{p},\;U_{p}=\frac{1}{2}Y_{p}\beta_{p}^{2}V_{p},\;U_{m}=\frac{1}{2}Y_{m}\beta_{m}^{2}V_{m},$$

where *β_m_ = β_p_* = *β* is the relative elongation (strain) of the structure, *V*_m_ and *V*_p_ are the volumes of the FM and PE layers, respectively. Let us represent the value of the energy, dissipated in each of the layers, through the mechanical quality factors of the layers:

(5) $$U_{m}^{diss}=\frac{U_{m}}{Q_{m}}\;\mathrm{and}\;U_{p}^{diss}=\frac{U_{p}}{Q_{p}}.$$

Then the following relation holds for the entire structure:

(6) $$\frac{U}{Q}=\frac{U_{m}+U_{p}}{Q}=\frac{U_{m}}{Q_{m}}+\frac{U_{p}}{Q_{p}}.$$

Substituting the expressions (4) for the strain energy of the layers, we obtain:

(7) $$Q=\frac{(Y_{m}b_{m}+Y_{p}b_{p})Q_{m}Q_{p}}{Y_{m}b_{m}Q_{m}+Y_{p}b_{p}Q_{p}}.$$

Analysis of Equation (7), taking into account the temperature dependences of the Young’s moduli (see Figure 2), the quality factors (see Figure 4), and the ratio of layer thicknesses, leads to the following conclusions:

- In structures with LGT layers and a thin FM layer, when *b*_p_ >> *b*_m_, the behavior of the *Q*(*T*)-dependence is determined by the PE layer, and the monotonic decrease in the quality factor with increasing temperature is caused by reduction of the mechanical quality factor of the langatate. For the LGT-Ni_T structure, the effect of a thick nickel layer, whose quality factor declines with the increasing temperature by a factor of 2, leads to a general reduction of the total quality factor in comparison to the LGT-Ni structure. Further, the loss of the total quality factor is pronounced more clearly with heating, since the quality factor of both layers decreases.
- The quality factor of structures with ceramic PZT layers is low and is determined by the small quality factor *Q*_p_ ≈ 100 of the PZT layer. In PZT structures with thin FM layers, the quality factor should remain approximately constant over the entire temperature range, since both the quality factor and the Young’s modulus of the PZT layer depend weakly on the temperature (see Figure 2 and Figure 3). This expectation is confirmed by the experiment. In the low-temperature range, the quality factor of PZT structures with a thick nickel layer grows with increasing temperature because of the increase in the quality factor of the Ni layer.

#### 3.3.3. Temperature Dependence of the Magnetoelectric Coefficient

To explain the results obtained, let us use the theory of the low-frequency ME effect in two-layer composite structures [43,44], which over the years has been repeatedly confirmed experimentally. The expression for the ME coefficient (see Equation (13) of reference [43]), corresponding to our experimental geometry, can be after some algebra reduced to the form of:

(8) $$\alpha_{E}=Q\frac{d_{31}\lambda^{\left(1\right)}}{\varepsilon \left[\frac{1}{Y_{m}}\frac{b_{p}}{b_{m}}+\frac{1}{Y_{p}}\right]-2d_{31}^{2}}.$$

Here *Q* takes into account the “enhancement” of the ME effect at the resonance frequency. Each of parameters in Equation (8), except of the thicknesses of the layers, depends on the temperature. Substituting the values of the experimental parameters into (8), we find that the second term in the denominator is always at least an order of magnitude smaller than the first term, therefore it can be neglected. Then Equation (8) takes the form of:

(9) $$\alpha_{E}=Q\frac{d_{31}\lambda^{\left(1\right)}}{\varepsilon \left(\frac{1}{Y_{m}}\frac{b_{p}}{b_{m}}+\frac{1}{Y_{p}}\right)}.$$

If the thickness of the PE layer is much larger than the thickness of the FM layer *b*_p_ >> *b*_m_, (in our case this condition holds for all structures except for the PZT-Ni-T composition) the expression (9) is further simplified to:

(10) $$\alpha_{E}=Q\frac{d_{31}\lambda^{\left(1\right)}Y_{m}}{\varepsilon }\frac{b_{m}}{b_{p}}.$$

Finally, taking into account that the Young’s moduli of all the materials in the investigated temperature range vary very weakly (by less than 4%, cf. Figure 4) in comparison to other parameters, we can neglect the dependence of *Y*_m_ on *T*. The final expression determining the temperature dependence of the ME interaction efficiency reads:

(11) $$\alpha_{E}(T)\sim Q(T)\frac{d_{31}(T)\lambda^{\left(1\right)}(T)}{\varepsilon (T)}.$$

Consequently, the variation of the ME coefficient with temperature is determined by the change in the acoustic quality factor *Q*, the piezoelectric modulus *d*_31_ and the relative permittivity *ε* of the PE layer, as well as the piezomagnetic modulus *λ*^(1)^ of the FM layer. For structures with an LGT layer, *d*_31_ in (11) should be replaced by *d*_11_.

On the basis of the obtained Equation (11), we can draw the following conclusions:

- For structures comprising LGT layers, whose PE modulus *d*_11_ and dielectric permittivity ε_11_ vary by 20% [29,31] and the quality factor *Q* is reduced threefold in the investigated temperature range, the acoustic quality factor of the entire structure plays crucial role in the temperature dependence of the ME coefficient. The thickening of the Ni layer in the LGT-Ni composite structure leads to an additional decrease in the quality factor and, consequently, to a stronger reduction the ME coefficient with increasing temperature. The smallest influence on the temperature dependence of *α*′*_E_*(*T*) is exercised by the piezomagnetic coefficient *λ*^(1)^ of a Ni or Metglas layer, which is a constitutive part of the structure.
- For structures comprising PZT layers, the particular form of the *α*′*_E_*(*T*)-dependence occurs as a result of competition of several effects. With an increase of temperature from 200 to 400 K, the dielectric constant *ε* of a PZT layer increases fourfold (see Figure 4), which should lead to a significant decrease of the ME signal. However, this is compensated by a threefold increase of the PE modulus *d*_31_ of PZT. As a result, the ratio *d*_31_/ε can only decrease by approximately 25% with increasing temperature. The quality factor of the PZT layer depends weakly on the temperature (see Figure 3). In this case, the behavior of the temperature dependence of the resonance ME coefficient of the PZT-Metglas structure will be determined by the temperature dependence of the piezomagnetic modulus *λ*^(1)^ of the FM layer. Indeed, the shapes of the curves α′(*T*) in Figure 13b and *λ*^(1)^(*T*) in Figure 10 are qualitatively the same. Similarly, for the PZT-Ni structure, the dependence of α′(*T*) is determined by the competition between two processes: a monotonic decrease of *d*_31_/ε with increasing temperature and a linear growth of *λ*^(1)^(*T*) for Ni (see Figure 11). The higher thermal stability of the ME coefficient of the PZT-Ni_T at temperatures *T* exceeding 320 K, in comparison with the PZT-Ni structure (Figure 3), can be explained by the growing contribution of the piezomagnetic modulus due to the larger thickness of the FM layer.

## 4. Temperature Characteristics of the Nonlinear Magnetoelectric Effect

The nonlinear ME effect comes into play in composite structures when the amplitude of the magnetic pump-field *h*_0_ is increased due to the nonlinear dependence of the magnetostriction of the FM layer on the DC field *H* [40,45]. The effect manifests itself in the form of generation of voltage harmonics with frequencies *f*, 2*f*, 3*f* etc., when it is excited by harmonic pump field *h*(*t*) with frequency *f*. Studies of nonlinear ME effects were carried out on the setup described above in the nonresonance regime. The frequency of the pump field was 120 Hz. The spectrum of the generated voltage was obtained using a spectrum analyzer SR 760.

**Figure 14:**
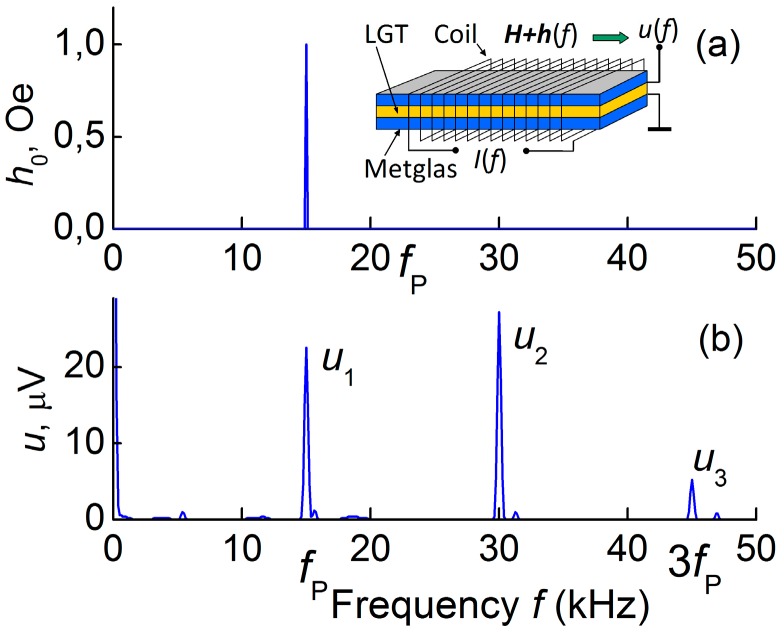
Frequency spectrum of the pump field (**a**) and the generated voltage (**b**) for a nonlinear ME effect in the LGT-Metglas structure.

As an example, Figure 14 shows a typical voltage spectrum for the nonlinear ME effect in the LGT-Metglas structure in the bias field *H* of 4 Oe and the pump field with *h*_0_ = 5 Oe. The spectral components with amplitudes *u*_1_, *u*_2_ and *u*_3_ are observed. Similar spectra were obtained at different temperatures in the range between 150 and 320 K.

**Figure 15:**
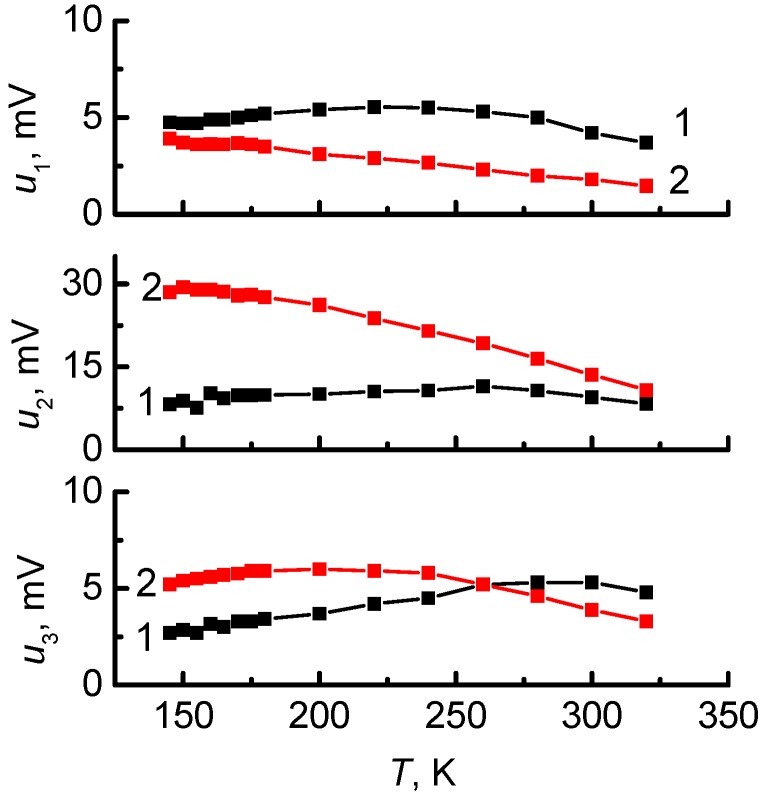
Temperature dependences of amplitudes of three harmonics of the ME signal for the LGT-Metglas structure at *H* = 4 Oe. Curve 1: *h*_0_ = 5 Oe; Curve 2: *h*_0_ = 15 Oe.

Figure 15 presents the temperature dependences of the amplitudes of the first three harmonics of the signal for two pump field amplitudes *h*_0_ = 5 and 15 Oe. Notice different behavior of temperature dependences of the harmonic amplitudes. For example, at *h*_0_ = 5 Oe, *u*_1_(*T*) first grows with the temperature, reaches a local maximum of 6 mV at *T* ≈ 220 K, and then decreases, the amplitude of the second harmonic commences to grow from *u*_2_ = 7 mV, reaches a local maximum at *T* ≈ 270 K, *u*_3_ monotonically increases from 2.5 mV at *T* = 150 K to 5 mV at *T* = 300 K. An increase in the pump field amplitude to 15 Oe has qualitatively changed the temperature dependences of the harmonic amplitudes. The harmonics *u*_1_ and *u*_2_ decrease monotonically approximately twofold when the temperature rises from 150 to 350 K, while *u*_3_ first increases with increasing temperature, reaches a local maximum at *T* ≈ 200 K, and then decreases.

Expression (11) is inapplicable for the interpretation of the obtained dependences of the ME signal harmonics, since it was obtained for small amplitudes *h*_0_, namely, for *h*_0_ << *H*_s_. Taking into account the weak temperature dependences of *d*_11_ and ε, the temperature dependences of the harmonics should be to a great extent determined by the value of the piezomagnetic coefficient:

(12) $$u_{n}(T)\sim \frac{d_{11}(T)q_{n}(h,H,T)}{\varepsilon (T)},$$

where *q_n_* is the piezomagnetic coefficient for the *n*-th harmonics. The magnitude of *q_n_* for large *h*_0_ can be either calculated numerically or obtained experimentally. The change of the qualitative behavior of the temperature dependences with an increase of the pump field from 5 to 15 Oe can be attributed to a change in the shape of the magnetostriction curve of the ferromagnetic material. For a detailed explanation of the relationships between the temperature dependences of ME voltage harmonics, additional investigations will be required for *h*_0_ ≈ *H*_s_.

## 5.Conclusions

Comprehensive experimental studies of the temperature characteristics of the resonance ME effect in composite structures comprising PE layers of single crystalline langatate material or a PE ceramic PZT material and FM layers of an amorphous alloy Metglas or nickel were carried out. It was found that in the temperature range *T* between 160 and 370 K the changes in the resonance frequency *f*_r_, the quality factor of the electromechanical resonance *Q* and the value of the ME coefficient α_E_ are determined by the temperature changes in the mechanical, dielectric, piezoelectric and magnetic characteristics of the constitutive layers of a structure. It is shown that the resonance frequency of the LGT-Metglas and LGT-Ni structures is practically independent of temperature and the resonance frequency of the PZT-Metglas and PZT-Ni structures is reduced by 2–6% due to decrease of the PZT rigidity with increasing temperature. In this case, an increase in the thickness of the FM layer leads to a larger reduction of *f*_r_ caused by a decrease in the rigidity of the Ni layer with increasing *T*. LGT-based structures have a high quality factor *Q* ≈ 5000, but it decreases two- to fivefold with increasing *T*. Composite structures comprising a PZT layer have a low quality factor *Q* ≈ 100 weakly dependent on *T*. The value of the ME coefficient *α*_E_ decreases monotonically with increasing temperature for all structures, except for the PZT-Metglas structure in the high-temperature range between 320 and 370 K. For LGT-based structures, the decrease of *α*_E_ is mainly due to a reduction of their acoustic quality factor. For PZT-based structures, the decrease of *α*_E_ can be explained by the increase of the PZT dielectric constant and reduction in the piezomagnetic modulus of the FM layer. It is demonstrated that by selecting the ratio of the thicknesses of PE and FM layers, it is possible to achieve temperature stabilization of the resonance frequency in structures with an LGT material and temperature stabilization of the ME coefficient α_E_ in structures with a PZT material. It is shown that the amplitudes of voltage harmonics generated due to nonlinearity of the ME interaction in composite structures have different temperature dependences whose behavior varies with the amplitude of the excitation field.

The results of the presented research can be useful for the design of composite ME structures with specified temperature characteristics, in particular for the development of temperature-stabilized ME devices. For example, it can be expected that these findings will be of particular importance for ME devices combining the sensing function with energy harvesting of mechanical vibrations which may lead to the development of magnetic-field sensor nodes for sustainable wireless sensor networks [45,46].

## Figures and Tables

**Table 1:** Physical properties of piezoelectric (PE) and ferromagnetic (FM) materials.

Material’s Notation	PZT-5	LGT	Ni	Metglas 2605S3A
Chemical composition	(Pb_0.95_Sr_0.05_) (Zr_0.53_Ti_0.47_)O	La_3_Ga_5.5_Ta_0.5_O_14_	Ni	FeBSiC
Mechanical quality factor, *Q*	90	10000	100	75
Young’s modulus, *Y* (10^10^ Pa)	6.2	26	20	10
Relative dielectric permittivity, *ε*	1100	20		
PE coefficient, *d* (pC/N)	*d*_31_ = 200	*d*_11_ = 5.1, *d*_14_ = 4.7		
Saturation magnetostriction, *λ*_S_ × 10^6^			−30	20
Saturation field, *H*_S_ (Oe)			~500	~60

PZT: lead zirconate titanate; LGT: langatate.

**Table 2:** Parameters of fabricated structures.

Sample		Thickness of FM Layer, (µm)	Thickness of PE Layer, (µm)	Length × Width, (mm × mm)
No.	Composition			
1	LGT-Metglas	20	500	20 × 5
2	LGT-Ni	50	500	20 × 5
3	PZT-Metglas	20	450	20 × 5
4	PZT-Ni	50	450	20 × 5
5	PZT-Ni_T	200	450	20 × 5
6	LGT-Ni_T	200	500	20 × 5

**Table 3:** Parameters of the resonance ME effect at *T* = 293 K.

Sample	*f*_r_ (kHz)	*Q*	*α*_E_ (V/cm × Oe)
LGT-Metglas	83.5	6000	450
LGT-Ni	84.2	5000	45.5
PZT-Metglas	62	120	135
PZT-Ni	65	100	3.0
PZT-Ni_T	111	110	10
LGT-Ni_T	94	170	10

*f*_r_: resonance frequency; *Q*: quality factor; *α*_E_: ME coefficient
